# Clinical significance of bone changes in osteoarthritis

**DOI:** 10.1186/ar3710

**Published:** 2012-03-08

**Authors:** Tuhina Neogi

**Affiliations:** 1 Section of Clinical Epidemiology Research and Training Unit, and Rheumatology, Department of Medicine, Boston University School of Medicine, Boston, MA, USA

## 

Knee OA is thought to be a largely mechanically-driven disease. Pertinent to this, bone is a dynamic tissue that adapts to loads by remodeling to meet its mechanical demands (Wolff's Law) [1,2]. As such, it is not surprising that bone changes, such as increased tibial plateau area and bone turnover, occur early in OA [3,4]. Recently, MRI-based 3D bone shape has been shown to track concurrently with OA onset, and to predict incidence of radiographic knee OA 12 months before its onset [5]. Further, in radiographically normal tibiofemoral knee joints, subchondral bone changes, such as bone marrow lesions (BMLs) on MRIs, are common.

Although MRIs allow direct visualization and morphologic evaluation of joint tissues not otherwise discernible on x-ray, their specific pathologies need to be examined by direct evaluation to gain further pathophysiologic insight. BMLs adjacent to the subchondral plate have been shown to have increased bone volume fraction and increased trabecular thickness, but reduced tissue mineral density (i.e., hypomineralized) [6], consistent with OA being associated with increased bone turnover. BMLs may render these areas mechanically compromised and susceptible to attrition. Indeed, BMLs are strongly associated with occurrence of subchondral bone attrition (SBA) [7]. Both subchondral bone abnormalities are associated with cartilage loss as well [8,9]. In keeping with knee OA being mechanically-driven, meniscal pathology, often the result of injury, is associated with new and enlarging BMLs [10]. Further, malalignment is associated with both BMLs and SBA [11,12].

The contributions of these structural abnormalities to the clinical manifestations of knee OA are becoming better understood. While it is widely thought that there is a structure-symptom discordance in knee OA, such observations do not take into account all of the potential factors that can contribute to between-person differences in the pain experience [13]. Recent work that used novel methodology to overcome this problem has demonstrated that pain fluctuation is associated with changes in BMLs, as well as synovitis and effusion [14]. SBA has also been associated with knee pain [15], but the relationship of osteophytes to pain has been conflicting. A challenge that remains in studying the specific contribution of pathologic features of OA to pain is the co-existence of multiple MRI abnormalities, making it difficult to identify individual pathologic features' effects.

Understanding the pathophysiologic sequences and consequences of OA pathology will guide rational therapeutic targeting. Importantly, rational treatment targets also require understanding what particular structures contribute to pain as pain is the reason patients seek medical care.
